# Paclitaxel Resistance Modulated by the Interaction between TRPS1 and AF178030.2 in Triple-Negative Breast Cancer

**DOI:** 10.1155/2022/6019975

**Published:** 2022-03-30

**Authors:** Tao Zhao, Tingrong Zhang, Yao Zhang, Bin Zhou, Xiangdong Lu

**Affiliations:** ^1^Department of Oncology, The Jiangyin Hospital Affiliated to Medical College of Southeast University, 163th Shoushan Road, Jiangyin, Wuxi, Jiangsu 214400, China; ^2^Department of Breast Armor Surgery, The Jiangyin Hospital Affiliated to Medical College of Southeast University, 163th Shoushan Road, Jiangyin, Wuxi, Jiangsu 214400, China

## Abstract

Paclitaxel is a chemotherapeutic agent that acts as an inhibitor of cellular mitosis and has been widely used in the treatment of triple-negative breast cancer (TNBC). However, paclitaxel resistance is one of the major reasons that contribute to the high failure rates of chemotherapy and the relapse of TNBC. Accumulating studies have demonstrated that long noncoding RNA (lncRNA) plays a role in the paclitaxel resistance and positively correlated with progression and metastasis of breast cancers. In the present study, microarray expression profile analysis of lncRNA was performed between paclitaxel-resistant TNBC cell line MDA-MB-231 and their parental cells. After verification with quantitative PCR, we identified that AF178030.2, an orphan lncRNA, was significantly upregulated in paclitaxel-resistant TNBC cells. Overexpression of AF178030.2 greatly attenuated the sensitivity of TNBC to paclitaxel, whereas knockdown of AF178030.2 enhanced the sensitivity of TNBC cells to paclitaxel. Furthermore, bioinformatic analysis and RNA binding protein immunoprecipitation assay reveal that AF178030.2 can directly bind with trichorhinophalangeal syndrome-1 (TRPS1), an oncogene in breast cancer, and downregulate its expression in paclitaxel-resistant TNBC cells. TRPS1 overexpression effectively increased the sensitivity of paclitaxel-resistant TNBC cells to paclitaxel. Taking together, high AF178030.2 expression contributed to paclitaxel resistance in TNBC through TRPS1 and poor clinical outcomes, which may provide a new treatment strategy for paclitaxel-resistant TNBC patients.

## 1. Introduction

Breast cancer is the most common malignant tumor in women worldwide. According to the latest statistics from the American Cancer Society, the mortality rate of breast cancer ranks only after lung cancer [1, 2]. Breast cancer can be divided into different types based on the expression of estrogen receptor (ER), progesterone receptor (PR), human epidermal growth factor receptor-2 (HER-2), and Ki-67 [1, 2]. Among them, triple-negative breast cancer (TNBC) refers to breast cancer with negative expression of ER, PR, and HER-2, accounting for about 10%–15% of breast cancer population [3, 4]. Compared with non-TNBC breast cancer, TNBC is characterized by strong invasion, high histological grade, easy recurrence, and metastasis and therefore has a worse prognosis [3, 4]. Moreover, targeted therapy and endocrine therapy are limited in TNBC due to its special molecular expression type [5]. So far, the treatment for patients with late stage TNBC is chemotherapy using taxanes and anthracyclines drugs [6, 7]. However, multiple cycles of chemotherapy will induce tumor cells to develop resistance to chemotherapeutic drugs, leading to treatment failure and tumor progression, which seriously affects the patient's quality of life and long-term survival rate. Therefore, how to overcome TNBC chemotherapy resistance is the key to improving the prognosis of TNBC patients.

Paclitaxel is a taxane compound and commonly used as a clinical chemotherapy drug for TNBC [8]. It is generally believed that paclitaxel can interfere with the normal polymerization and depolymerization of microtubules by binding to tubulin, thereby blocking the cell cycle in the G2/M phase and initiating cell apoptosis [9, 10]. Clinical data have demonstrated that, during the long-term paclitaxel chemotherapy, nearly half of the patients showed obvious paclitaxel resistance and tumor progressed [8, 11]. Therefore, drug resistance is the main challenge of paclitaxel treatment in TNBC [11]. In-depth exploration of the molecular mechanism of paclitaxel resistance to discover new intervention targets is urgent and necessary to effectively overcome paclitaxel chemotherapy resistance in clinical practice. Long noncoding RNAs (lncRNAs) are defined as noncoding RNA longer than 200 nucleotides and found to be important regulators of diverse biological processes, and their expression is altered in multiple pathologies including cancer [12, 13]. Recently, accumulating studies have demonstrated the important roles of lncRNAs in the chemotherapy resistance of a variety of tumors [14, 15]. Therefore, identifying the lncRNAs that are involved in the paclitaxel resistance may shed a new light on overcoming paclitaxel chemotherapy resistance.

In the present study, we performed microarray expression profile analysis in paclitaxel-resistant MDA-MB-231 cells and identified an orphan lncRNA, AF178030.2, which was extensively upregulated in paclitaxel-resistant cells and was able to mediate the sensitivity and resistance to paclitaxel by downregulating the expression of trichorhinophalangeal syndrome-1 (TRPS1), an important regulator in epithelial-mesenchymal transition, which was found to be one of the main reasons to induce drug resistance in many cancers.

## 2. Material and Methods

### 2.1. Transient Gene Transfection

The plasmid expressing AF178030.2 or TRPS1 coding region was constructed by Gibson assembly using lentivirus vector (pCDHL). To overexpress AF178030.2 or TRPS1, the plasmids with AF178030.2 or TRPS1 coding region were transiently transfected by TNBC cell lines using Lipofectamine™ 3000 (Invitrogen, USA) according to the manufacturer's instructions. The vectors of AF178030.2 were also transfected by HEK293T cells for lentivirus packaging. MDA-MB-231 were then infected by HEK293T cell supernatant and were selected in media supplemented with 5 *μ*g/ml of puromycin.

To knock down AF178030.2 expression, we obtained two AF178030.2 siRNA constructs (5′-TGC TGT TCA ATC AGA TAT T-3′) and a negative control scrambled siRNA (NC) construct from GenePharma (China) and then transiently transfected TNBC cell lines using Lipofectamine™ 3000 (Invitrogen, USA) according to the manufacturer's instructions.

### 2.2. Cell Culture and Establishment of Paclitaxel-Resistant Cell Lines

The human cell lines of TNBC, MDA-MB-231, and MDA-MB-436 were obtained from The Cell Bank of Type Culture Collection of Chinese Academy of Sciences (Shanghai, China) and cultured with Dulbecco's Modified Eagle's medium (Gibco, Thermo Fisher Scientific, Inc.) supplemented with 10% fetal bovine serum and 1 × penicillin streptomycin (Corning) in a 37°C incubator with an atmosphere of 5% CO_2_ and 95% air. MDA-MB-231 and MDA-MB-436 paclitaxel-resistant cell lines were established by gradual administration of increasing concentration of paclitaxel (Sigma-Aldrich, USA) into culture medium from 1 nM to 100 nM for 6 months. The medium with paclitaxel was changed every two days.

### 2.3. Microarray Expression Profile Analysis

Total RNA was isolated from MDA-MB-231 paclitaxel-resistant cells and their parental cells using Trizol reagent. The human LncRNA microarray V2.0 (Arraystar Co., USA) containing lncRNAs was used in this study. About 33,000 lncRNAs were collected from different data sources including NCBI RefSeq, RNAdb, UCSC, and UCRs. Each lncRNA array was composed of more than 60,000 distinct probes (60 mers), and each lncRNA was represented by 1∼5 probes. The microarray hybridization and bioinformatic analysis was performed by KangChang Biotech, Co. (Shanghai, China).

### 2.4. Cell Growth Assay

Cell growth rate of MDA-MB-231 and MDA-MB-436 paclitaxel-resistant cells and their parental cells was determined by cell proliferation assay via real-time cell impedance analysis (RTCA). Briefly, the cell proliferation rates were monitored dynamically by the xCELLigence system (Roche Applied Science) according to the manufacturer's instructions. The impedance was indicated as cell index. RTCA software was applied to analyze the measurements.

### 2.5. MTT Assay

Cell proliferation was determined by a 3-(4,5-demerthylthiazol-2-yl)-2,5-diphenyltetrazolium bromide (MTT) assay according to the manufacturer's instruction. Briefly, cells were seeded in 96-well culture plates at a density of 4 × 10^3^ cells per well. After treatment with various drugs for indicated periods of time, the medium was changed. 5 mg/ml MTT was then added to each well and incubated for 4 h at 37°C. Subsequently, the supernatant was discarded and 150 *μ*L dimethyl sulfoxide was added to dissolve intracellular formazan crystals. The cell viability in each well was determined by measuring the absorbance at 490 nm using a microplate spectrophotometer. Each cell viability assay was performed in triplicate.

### 2.6. Colony Formation Assay

Colony formation assay was performed to determine the survival ability of a single cell to grow into a colony. MDA-MB-231 and MDA-MB-436 paclitaxel-resistant cells were seeded in 6-well plates (500 cells/well) with 4 ml complete medium. After one week of culture, the colonies were washed with phosphate buffer saline and fixed with methanol at room temperature for 30 min. After staining with 0.1% crystal violet dye for 30 min, the cells were placed under a microscope and the images of the stained colonies were captured. Finally, the number of colonies was counted from these images.

### 2.7. Gene Ontology Analysis

Hierarchical clustering analysis of genes was carried out, which were differentially expressed between naïve and resistant cell lines. Potential targets of mRNAs were analyzed by Gene Ontology (GO). The biological process (BP), cellular component (CC), and molecular function (MF) of potential targets were clustered based on ClusterProfiler package in R software (version: 3.18.0). In the enrichment result, *P* < 0.05 or FDR <0.05 is considered to be enriched to a meaningful pathway.

### 2.8. Quantitative Real-Time PCR

Total RNA was extracted using Trizol reagent according to the manufacturer's instruction. 2 *μ*g total RNA was reverse-transcribed into the first-strand cDNA using Oligo dT primer and random primer. The PCR primers for amplification were as follows: TRPS1 sense-5′ CGC GAA GGC TCC TTT GAT ATT3′, antisense-5′ GCG AGA GAG CAA TCG AGA GG3′; AF178030.2 sense-5′ CTC GAC GTC ACT TCT TCC ACA 3′, antisense-5′ GGC TCC TTT TCT CCA CCA GAA3′; BC015064 sense-5′ CAG GAT ACC AGT GCC CTT CA3′, antisense-5′ CGT AGC AGC TAT GCA GCT TG 3′; AM259138 sense-5′ TTG GTA CAG CAG ACT ACG CA 3′, antisense-5′ GAC TAG TTT CTC GCG CAC AG 3′; SND1 sense-5′ GAC TGC TGT CCT TAC AGG GG 3′, antisense-5′ TGG CTG GGG TTG CAT AAC TC3′; AK129540 sense-5′ TTC CAT TCC ATC GTC TGG GT 3′, antisense-5′ TGA AAG GAA GCA GGA GAG CA 3′; AC073934.6 sense-5′ CCA CTC GAC TTC CGA CAT T 3′, antisense-5′ CCT CCA GCT TCT TGA TCT TTC3′; NR_024579 sense-5′ CCC TCA TTC CTC CAG AGC TT 3′, antisense-5′ TAG CAT GAT CCC AGC TAC CC 3′; NR_023391 sense-5′ CTT CAT TGA TGC GCT TGT GC 3′, antisense-5′ ACA CTG AGG CTT GAT CAG ACA 3.

The expression level of mRNA was measured based on the threshold cycle (Ct), and relative expression levels were calculated as 2^−((Ct of mRNA)–(Ct of GAPDH))^ after normalization to GAPDH expression.

### 2.9. Western Blot

Total proteins were extracted from cells by radioimmunoprecipitation assay buffer (RIPA buffer). The protein concentration was determined by bicinchoninic acid (BCA) method. 20 *μ*g of total protein was separated by SDS-PAGE and was then transferred onto PVDF membrane. After blockage with 5% nonfat milk, the PVDF membrane was incubated with primary antibodies (anti-TRPS1 antibody, ab125197; anti-*β*-actin antibody, ab8226) overnight at 4°C. After washing, the membrane was further incubated with HRP-conjugated secondary antibody. The signal was developed by film. Relative protein expression levels were quantified by scanning densitometry.

### 2.10. RNA Binding Protein Immunoprecipitation Assay

The cells were lysed in low-sucrose buffer containing 0.3 M sucrose, 5 mM CaCl2, 5 mM MgAc2, 0.1 mM EDTA, 50 mM HEPES, 1 mM DTT, and 0.1% Triton X-100 on ice. The lysate was then cross-linked by 1% formaldehyde. After washing twice with the following buffer (150 mM NaCl, 50 mM Tris, 20 mM EDTA, 0.5% NP-40, and 1% Triton-X-100) and incubated in lysis buffer (100 mM Tris-HCl, 20 mM EDTA, and 2% SDS) for 10 min, the lysate was precleared with Dynabeads protein A (Invitrogen) and then incubated with anti-TRPS1 antibody (ab125197, Abcam) for additional 2 hours at 4°C. After washing, the RNA was eluted from beads and purified by an miRNeasy Mini Kit (Qiagen). During the procedure, RNAse inhibitor (Invitrogen) and protease inhibitors (Roche, cocktail) were added to all solutions. Finally, the purified RNA was reversed-transcribed for qRT-PCR analysis.

### 2.11. Statistical Analysis

The data were expressed as mean ± standard deviation, unless otherwise stated. All statistical analyses were performed using the Prism GraphPad statistical software. *T* test and a one-way ANOVA were used for the statistical analysis. *P*=0.0416 were considered significant.

## 3. Results

### 3.1. Microarray Expression Profile Analysis of lncRNA in Paclitaxel-Resistant TNBC Cells

**Figure 1:**
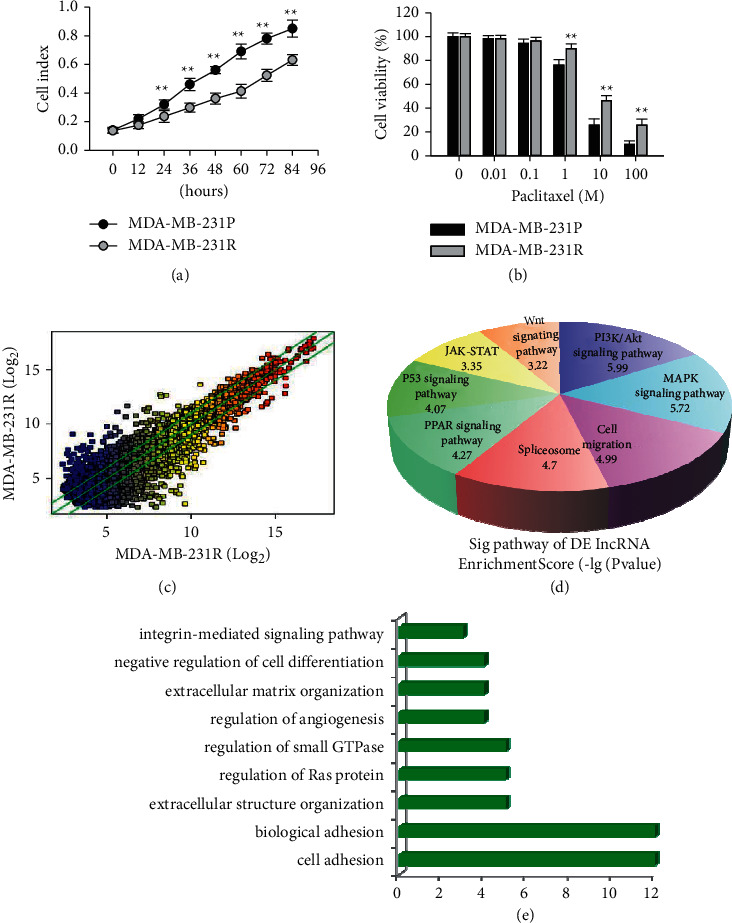
Microarray expression profile analysis of lncRNA in paclitaxel-resistant TNBC cells. (a) Comparison of the growth rate between the parental cells (MDA-MB-231P) and the paclitaxel-resistant cells (MDA-MB-231R). (b) MTT assay showing the comparison of the cell viability between MDA-MB-231P and MDA-MB-231R after treatment with different concentration of paclitaxel, indicating the paclitaxel sensitivity of MDA-MB-231R cells. ^*∗∗*^*P* < 0.01 versus MDA-MB-231P. *n* = 5. (c) Comparison of the lncRNA expression levels between MDA-MB-231R and MDA-MB-231P. Log2 scale was used in *X*- and *Y*-axis. (d) Gene Ontology analysis showing the predicted target genes of differentially expressed lncRNAs. (e) Pathway analysis showing the predicted target genes of differentially expressed lncRNAs.

To establish a cell model of paclitaxel-resistant TNBC, MDA-MB-231 cell line was cultured in a continuous treatment with a gradually increasing concentration of paclitaxel (1–100 nM) for 6 months. Compared with their parental cells (MDA-MB-231P), paclitaxel-resistant cells (MDA-MB-231R) grew significantly slower (Figure 1(a)). MTT assay was used to verify the establishment of paclitaxel-resistant TNBC. As shown in Figure 1(b), the sensitivity to paclitaxel of MDA-MB-231R was dramatically reduced, and MDA-MB-231R survived well in 1 *μ*M paclitaxel. Similar results were found using another TNBC cell line, MDA-MB-436 (Supplemental Figure 1).

To search for the key lncRNA that us involved in the paclitaxel resistance, we first performed microarray expression profile analysis to identify some lncRNAs that were differentially expressed in the paclitaxel resistance. Based on the data in Figure 1(c), the lncRNA expression levels between MDA-MB-231R and MDA-MB-231P were compared and showed 1025 upregulated lncRNAs and 656 downregulated lncRNAs that were significantly differentially expressed (>2-fold change). To generally understand potential roles of these differentially expressed lncRNAs in the paclitaxel resistance, Gene Ontology (GO) analysis and pathway analysis were performed to determine the predicted target genes of differentially expressed lncRNAs attribute in biological processes and molecular functions. As shown in Figures 1(d) and 1(e), the potential target genes of high and low expression of lncRNA in paclitaxel-resistant MDA-MB-231R cells mainly involved the processes of drug resistance, invasion, and metastasis, indicating the important roles of lncRNA in the paclitaxel resistance of TNBC.

### 3.2. Upregulation of lncRNA AF178030.2 and Its Clinical Outcome in Paclitaxel-Resistant TNBC Patients

**Figure 2:**
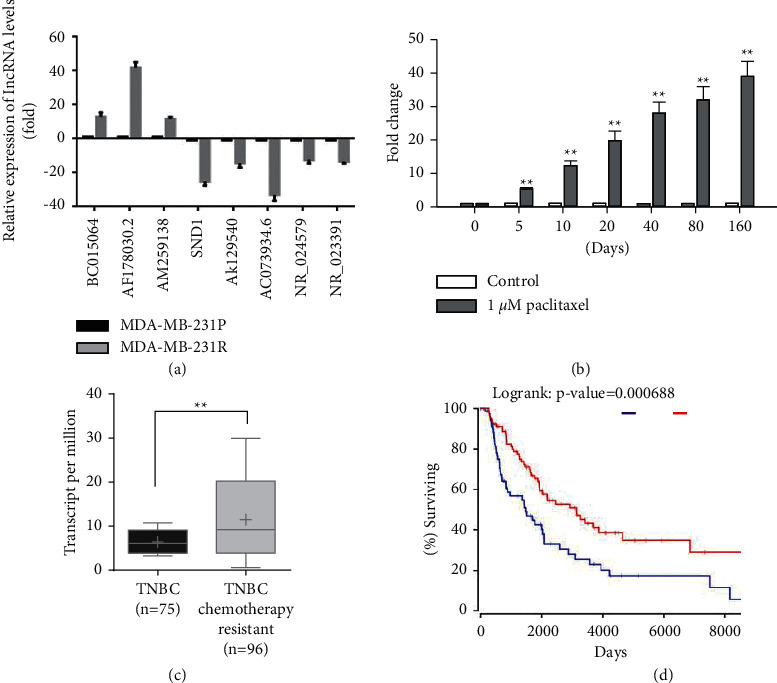
Upregulation of lncRNA AF178030.2 and its clinical outcome in paclitaxel-resistant TNBC patients. (a) Quantitative real-time PCR showing eight lncRNAs expression, including BC015064, AF178030.2, AM259138, SND1, AK129540, AC073934.6, NR_024579, and NR_023391 between MDA-MB-231R and MDA-MB-231P cells. (b) The expression level of AF178030.2 was determined by quantitative real-time PCR after different days treatment with 1 *μ*M paclitaxel in MDA-MB-231 cells. ^*∗∗*^*P* < 0.01 versus control group. (c) Comparison of AF178030.2 expression level in chemotherapy-resistant patients of TNBC compared to chemotherapy-sensitive patients; all the data were retrieved from the METABRIC database. (d). Kaplan–Meier survival curves of TNBC patients undergoing chemotherapy with low and high AF178030.2 expression based on METABRIC database (*P*=0.0416).

Next, quantitative real-time PCR was employed to verify the expression level of lncRNAs that have >10-fold change in the microarray expression profile analysis, including BC015064, AF178030.2, AM259138, SND1, AK129540, AC073934.6, NR_024579, and NR_023391, using another MDA-MB-231R and MDA-MB-231P samples. We found that the fold changes of these lncRNAs was quite consistent, with AF178030.2 having highest fold change (Figure 2(a)). Therefore, we focused on the lncRNA AF178030.2 in the following experiments. Indeed, the expression level of AF178030.2 was elevated by paclitaxel treatment (1 *μ*M) in a time-dependent manner in MDA-MB-231 and MDA-MB-436 cells (Figure 2(b) and Supplemental Figure 2), indicating that AF178030.2 may mediate the paclitaxel resistance of MDA-MB-231 and MDA-MB-436 cells. Furthermore, we retrieved the expression data of AF178030.2 from the TCGA. As shown in Figure 2(c), the AF178030.2 expression was significantly upregulated in chemotherapy-resistant patients of TNBC breast cancer than chemotherapy-sensitive patients. More importantly, our analysis revealed that higher AF178030.2 expression was associated with shorter overall survival of TNBC breast cancer patients (Figure 2(d)).

### 3.3. Overexpression of AF178030.2 Attenuated the Sensitivity of TNBC to Paclitaxel, Whereas Knockdown of AF178030.2 Enhanced the Sensitivity of TNBC Cells to Paclitaxel

**Figure 3:**
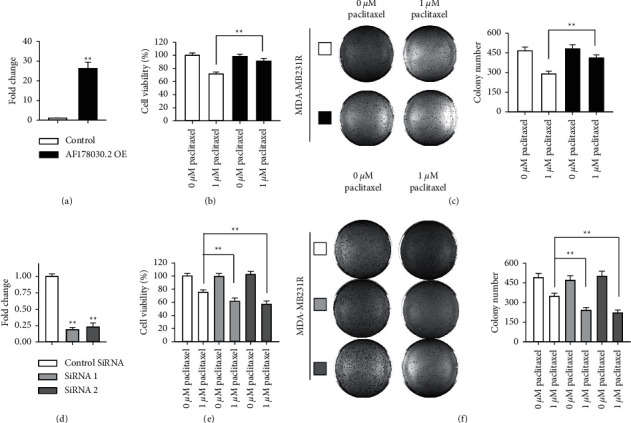
Overexpression of AF178030.2 attenuated the sensitivity of TNBC to paclitaxel, whereas knockdown of AF178030.2 enhanced the sensitivity of TNBC cells to paclitaxel. (a) Quantitative PCR result showing the expression level of AF178030.2. (b) MTT assay showing the cell viability. (c) Left: representative results of colony formation assay; right: the collective data of colony number after AF178030.2 overexpression. (d) Quantitative PCR result showing the knockdown effect of AF178030.2 by two separate siRNAs. (e) MTT assay showing the cell viability after AF178030.2 knockdown. (f) Colony formation assay showing the colony number after AF178030.2 knockdown. ^*∗∗*^*P* < 0.01. *n* = 5.

To investigate the role of lncRNA AF178030.2 in paclitaxel resistance of TNBC, the construct for AF178030.2 expression was generated and transfected into MDA-MB-231. Quantitatively PCR results showed that the expression level of AF178030.2 was overexpressed ∼26 folds after 3 days (Figure 3(a)). MTT assay was used to determine the cell proliferation of MDA-MB-231 after overexpression of AF178030.2. As shown in Figure 3(b), AF178030.2 overexpression in MDA-MB-231 significantly attenuated the inhibitory effect of 1 *μ*M paclitaxel in cell proliferation. Moreover, the colony formation assay also showed a similar result, in which the paclitaxel-mediated inhibition of cell growth was partly eliminated by AF178030.2 overexpression (Figure 3(c)). Similar results were found using another TNBC cell line, MDA-MB-436 (Supplemental Figure 3). There results suggested that the sensitivity of paclitaxel was reduced by AF178030.2 overexpression in MDA-MB-231 and MDA-MB-436 cells.

The complementary experiments were also done by knocking down AF178030.2 expression using shRNA. Compared to the control shRNA, the expression level of AF178030.2 in MDA-MB-231 cells was reduced to ∼20% in two different shRNAs that target AF178030.2 (Figure 3(d)). MTT assay and colony formation assay both showed that knockdown of AF178030.2 expression significantly increased paclitaxel-induced cell proliferation and growth (Figures 3(e) and 3(f)). Taken together, these results indicated that lncRNA AF178030.2 mediated the sensitivity and resistance of paclitaxel in MDA-MB-231 cells.

### 3.4. TRPS1 is a Binding Target of AF178030.2 and Regulates the Sensitivity of TNBC Cells to Paclitaxel

**Figure 4:**
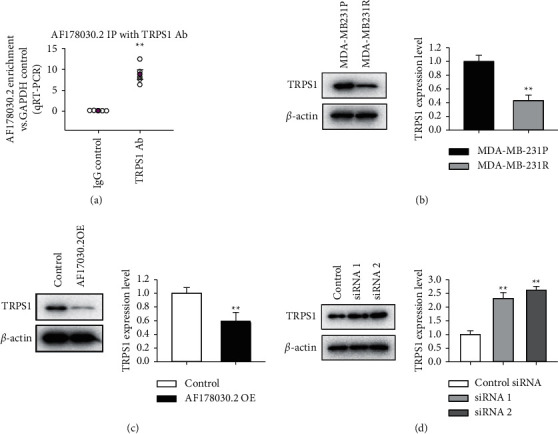
TRPS1 is a binding target of AF178030.2. (a) RNA binding protein immunoprecipitation assay result showing the interaction between AF178030.2 and TRPS1. Upon immunoprecipitating TRPS1, the presence of AF178030.2 was tested by quantitative RT-PCR, and the enrichment versus the housekeeping mRNA GAPDH was calculated. Ab: antibody; IgG: immunoglobulin G. *n* = 4. (b) Comparison of TRPS1 protein level between MDA-MB-231R and MDA-MB-231P cells. (c) TRPS1 protein level after AF178030.2 overexpression. (d) TRPS1 protein level after knockdown of AF178030.2. ^*∗∗*^*P* < 0.01 versus control group. *n* = 5.

To investigate the mechanisms underlying the regulation of AF178030.2 in paclitaxel resistance, bioinformatic analysis was performed to predict the potential target of AF178030.2. Interestingly, we found that AF178030.2 can directly bind with trichorhinophalangeal syndrome-1 (TRPS1), an important regulator in epithelial-mesenchymal transition, which was found to be one of the main reasons to induce drug resistance in many cancers [16, 17]. RNA binding protein immunoprecipitation assay result verified the interaction between AF178030.2 and TRPS1 (Figure 4(a)). Indeed, the expression level of TRPS1 was downregulated in MDA-MB-231R cells, compared with MDA-MB-231P cells (Figure 4(b)). Overexpression of AF178030.2 greatly reduced the TRPS1 expression (Figure 4(c)), whereas knockdown of AF178030.2 increase has an opposite effect (Figure 4(d)). Similar results were found using another TNBC cell line, MDA-MB-436 (Supplemental Figure 4). These results indicated that AF178030.2 mediate the sensitivity of paclitaxel in MDA-MB-231 and MDA-MB-436 cells by interacting with TRPS1.

**Figure 5:**
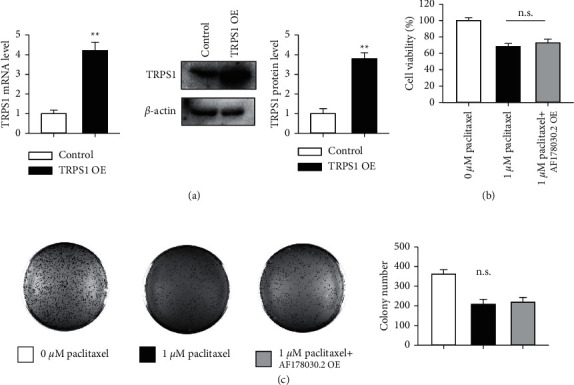
Overexpression of TRPS1 eliminated the effect of AF178030.2 on the sensitivity of TNBC cells to paclitaxel. (a) Quantitative RT-PCR and Western blot results showing the overexpression of TRPS1 in MDA-MB-231 cells. ^*∗∗*^*P* < 0.01 versus control group. (b) MTT assay showing the effect of TRPS1 overexpression on the cell viability. (c) Colony formation assay showing the colony number after TRPS1 overexpression. *n* = 5.

To test this hypothesis, we established an MDA-MB-231 cell line that stably overexpressed TRPS1 by lentivirus infection and puromycin selection. The mRNA and protein expression of TRPS1 was examined by real-time PCR and Western blot, respectively. As shown in Figure 5(a), TRPS1 expression was elevated by ∼4 folds both in mRNA and protein levels. Then, this cell line was challenged by AF178030.2 overexpression and 1 *μ*M paclitaxel treatment. Interestingly, MTT assay showed that, in this cell line, AF178030.2 overexpression cannot attenuate the inhibitory effect of paclitaxel in cell proliferation (Figure 5(b)). Consistently, AF178030.2 overexpression also cannot affect the paclitaxel-mediated inhibition of cell growth detected by the colony formation assay (Figure 5(c)). These results were confirmed by using another TNBC cell line, MDA-MB-436 (Supplemental Figure 5), suggesting that TRPS1 was a downstream target of AF178030.2 that mediated the sensitivity of paclitaxel in MDA-MB-231 and MDA-MB-436 cells.

## 4. Discussion

In the present study, we first established a TNBC cell model with paclitaxel resistance in MDA-MB-231 cells. Combining microarray expression profile analysis and lncRNA, gain/loss-of-function experiments, and functional assays, we identified a novel lncRNA, AF178030.2, which is an important regulator during the process of paclitaxel resistance. Furthermore, TRPS1 was found to be a target of AF178030.2 that regulates the sensitivity of TNBC cells to paclitaxel. The AF178030.2-TRPS1 axis may shed a new light on the underlying mechanism of paclitaxel resistance in TNBC.

Compared with non-TNBC, TNBC has a poorer prognosis due to its strong invasiveness, high histological grade, and easy recurrence and metastasis [2–4]. TNBC is more common in young women. Paclitaxel is the most used clinical chemotherapy drug for TNBC [7, 9]. The main mechanism of action is to interfere with the normal polymerization and depolymerization of microtubules by binding to tubulin, thereby arresting the cell cycle in the G2/M phase and then initiating cell apoptosis [9, 10]. Clinical data showed that when paclitaxel is used as a first-line chemotherapy drug, its effective rate is about 50%; when used as a second-line or third-line chemotherapy drug, its effective rate drops to 20%–30%, and it is prone to develop chemotherapy resistance after about 6 to 10 months. The tumor then progressed, and nearly half of the patients showed obvious paclitaxel resistance [8]. Therefore, drug resistance is the main reason for the failure of paclitaxel treatment [11]. It is generally believed that the resistance mechanism of paclitaxel may include (1) abnormal expression of drug transporters such as ABC transporter, P-glycoprotein, and multidrug resistance protein [18, 19]; (2) changes in drug targets such as tubulin [20]; (3) changes in the expression of apoptosis regulatory proteins such as p53 and Bcl-2 [21, 22]; and (4) abnormal drug metabolism/inactivation [23]. However, the strategies taken for the above drug resistance mechanisms have not improved the clinical paclitaxel resistance problem well. Therefore, in-depth exploration of the molecular mechanism of paclitaxel resistance to discover new intervention targets is necessary to effectively overcome paclitaxel chemotherapy resistance in clinical practice. Here, we found 1025 upregulated lncRNAs and 656 downregulated lncRNAs that were significantly differentially expressed in the paclitaxel-resistant cells, and the potential target genes of these differentially expressed lncRNA mainly involved the processes of drug resistance, invasion, and metastasis, suggesting the important roles of lncRNA in the paclitaxel resistance of TNBC.

Our microarray expression profile analysis showed that lncRNA178030.2 is a highly expressed lncRNA in paclitaxel-resistant MDA-MB-231 cells, which was further verified by quantitative RT-PCR assay. LncRNA178030.2 was also significantly upregulated in TNBC chemotherapy resistant tissues and highly associated with the poor prognosis of TNBC. LncRNA178030.2 has a full length of 402 nt and is an antisense transcript of the intron of the TRPS1 gene, suggesting that TRPS1 may be one of the targets of lncRNA178030.2. In this study, overexpression of lncRNA178030.2 significantly attenuated the inhibitory effect of 1 *μ*M paclitaxel in cell proliferation of MDA-MB-231, while knockdown of lncRNA178030.2 had an opposite effect, suggesting that lncRNA178030.2 mediated the process of paclitaxel resistance of breast cancer cells.

TRPS1 is a newly discovered member of the GATA transcription factor family. The human TRPS1 gene is located on chromosome 8q24.1 and encodes a transcriptional regulator composed of 9 zinc fingers. TRPS1 protein contains 1 GATA-type DNA binding domain, 2 potential nuclear localization signals, and 2 C-terminal zinc finger domains [16]. The TRPS1 gene is highly conservative, and its encoded product TRPS1 is widely expressed in human prostate, testis, ovary, kidney, lung, and breast tissues and mainly exists in the nucleus [16, 24, 25]. As a transcription factor, TRPS1 can both promote and inhibit the expression of target genes [26]. Accumulating studies have shown [16, 27, 28] that TRPS1 is an important regulator of epithelial-mesenchymal transition (EMT) by regulating the expression of key regulators of EMT. It has been demonstrated that EMT is one of the important mechanisms for inducing drug resistance in tumors [17, 29]. Here, RNA binding protein immunoprecipitation assay result verified the interaction between AF178030.2 and TRPS1. Importantly, the expression level of TRPS1 can be regulated by AF178030.2. Gain-of-function experiments showed that TRPS1 overexpression disturbed the effect of AF178030.2 on the sensitivity of TNBC cells to paclitaxel, suggesting that AF178030.2-TRPS1 axis is an important regulator during the paclitaxel resistance process.

In summary, lncRNA AF178030.2 was identified to regulate the paclitaxel resistance by targeting TRPS1, which may provide a new treatment strategy of paclitaxel-resistant TNBC patients.

## Data Availability

All the data can be provided upon request.

## Supplementary Materials

Supplementary Materials Supplemental Figure 1: establishment of paclitaxel sensitivity of MDA-MB-436R cells. Supplemental Figure 2: the expression level of AF178030.2 was determined by quantitative real-time PCR after different days treatment with 1 *μ*M paclitaxel in MDA-MB-436 cells. Supplemental Figure 3: overexpression of AF178030.2 attenuated the sensitivity of TNBC to paclitaxel in MDA-MB-436 cells. Supplemental Figure 4: AF178030.2 regulated the expression level of TRPS1in MDA-MB-436 cells. Supplemental Figure 5: overexpression of TRPS1 eliminated the effect of AF178030.2 on the sensitivity of TNBC cells to paclitaxel.
