# Supplementary material for: Paclitaxel Resistance Modulated by the Interaction between TRPS1 and AF178030.2 in Triple-Negative Breast Cancer
Source: Evid Based Complement Alternat Med. 2022 Mar 30;2022:6019975. doi: 10.1155/2022/6019975 (PMC8986375; doi:10.1155/2022/6019975)
Supplement: Supplementary Materials — Supplemental Figure 1: establishment of paclitaxel sensitivity of MDA-MB-436R cells. Supplemental Figure 2: the expression level of AF178030.2 was determined by quantitative real-time PCR after different days treatment with 1 μM paclitaxel in MDA-MB-436 cells. Supplemental Figure 3: overexpression of AF178030.2 attenuated the sensitivity of TNBC to paclitaxel in MDA-MB-436 cells. Supplemental Figure 4: AF178030.2 regulated the expression level of TRPS1in MDA-MB-436 cells. Supplemental Figure 5: overexpression of TRPS1 eliminated the effect of AF178030.2 on the sensitivity of TNBC cells to paclitaxel. [file 6019975.f1.pdf]

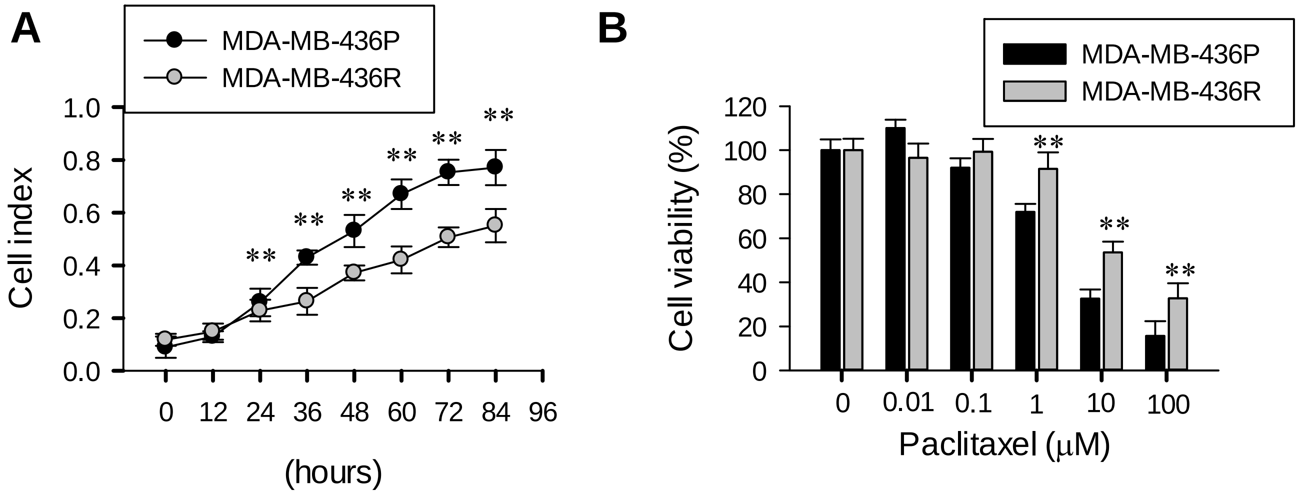

Supplemental Figure 1. Establishment of paclitaxel sensitivity of MDA-MB-436R cells. A. Comparison of the growth rate between the parental cells (MDA-MB-436P) and the paclitaxel-resistant cells (MDA-MB-436R). B. MTT assay showing the comparison of the cell viability between MDA-MB-436P and MDA-MB-436R after treatment with different concentration of paclitaxel, indicating the paclitaxel sensitivity of MDA-MB-436R cells. ***P<0.01* versus MDA-MB-436P. n=5.

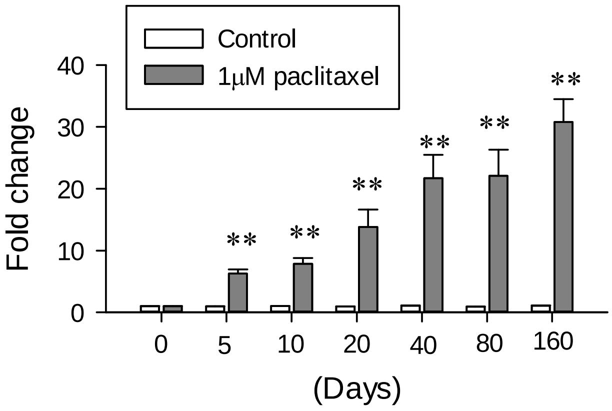

Supplemental Figure 2. The expression level of AF178030.2 was determined by quantitative real-time PCR after different days treatment with 1 μM paclitaxel in MDA-MB-436 cells. ***P<0.01* versus control group.

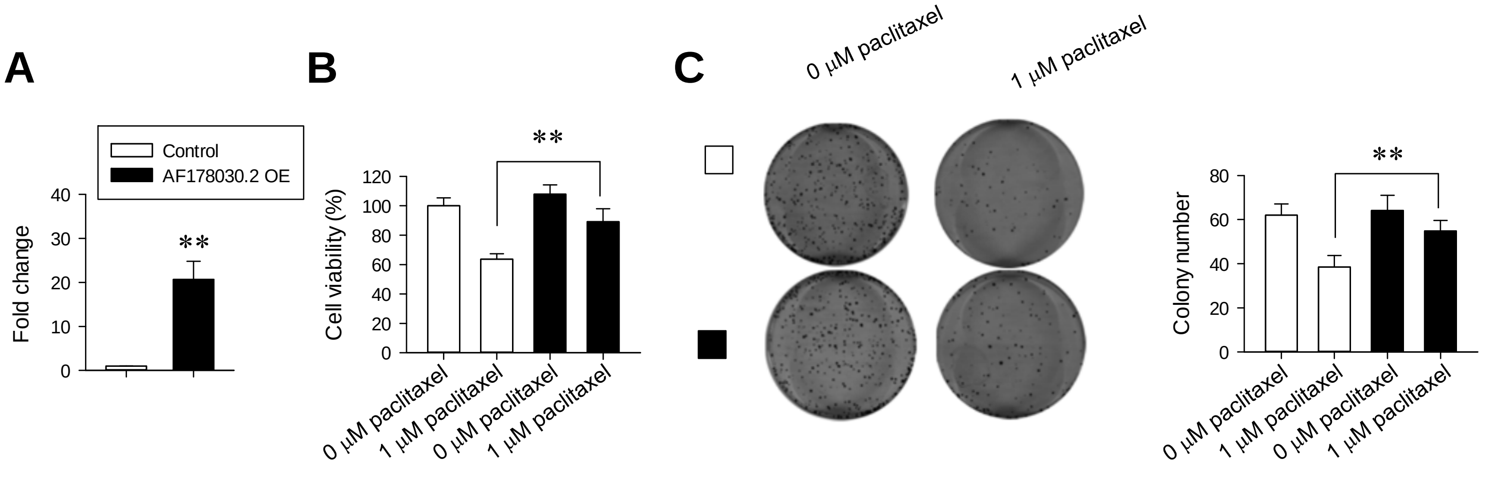

Supplemental Figure 3. Overexpression of AF178030.2 attenuated the sensitivity of TNBC to paclitaxel in MDA-MB-436 cells. A. Quantitatively PCR result showing the expression level of AF178030.2. B. MTT assay showing the cell viability. C. Left: representative results of colony formation assay; Right: the collective data of colony number after AF178030.2 overexpression.

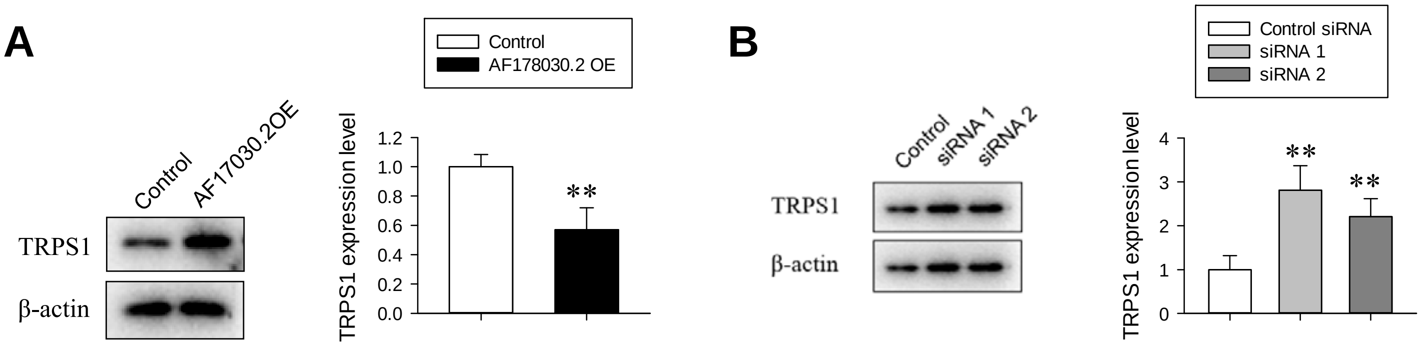

Supplemental Figure 4. AF178030.2 regulated the expression level of TRPS1in MDA-MB-436 cells. A. TRPS1 protein level after AF178030.2 overexpression. B. TRPS1 protein level after knockdown of AF178030.2. **P<0.01 versus control group. n=5.

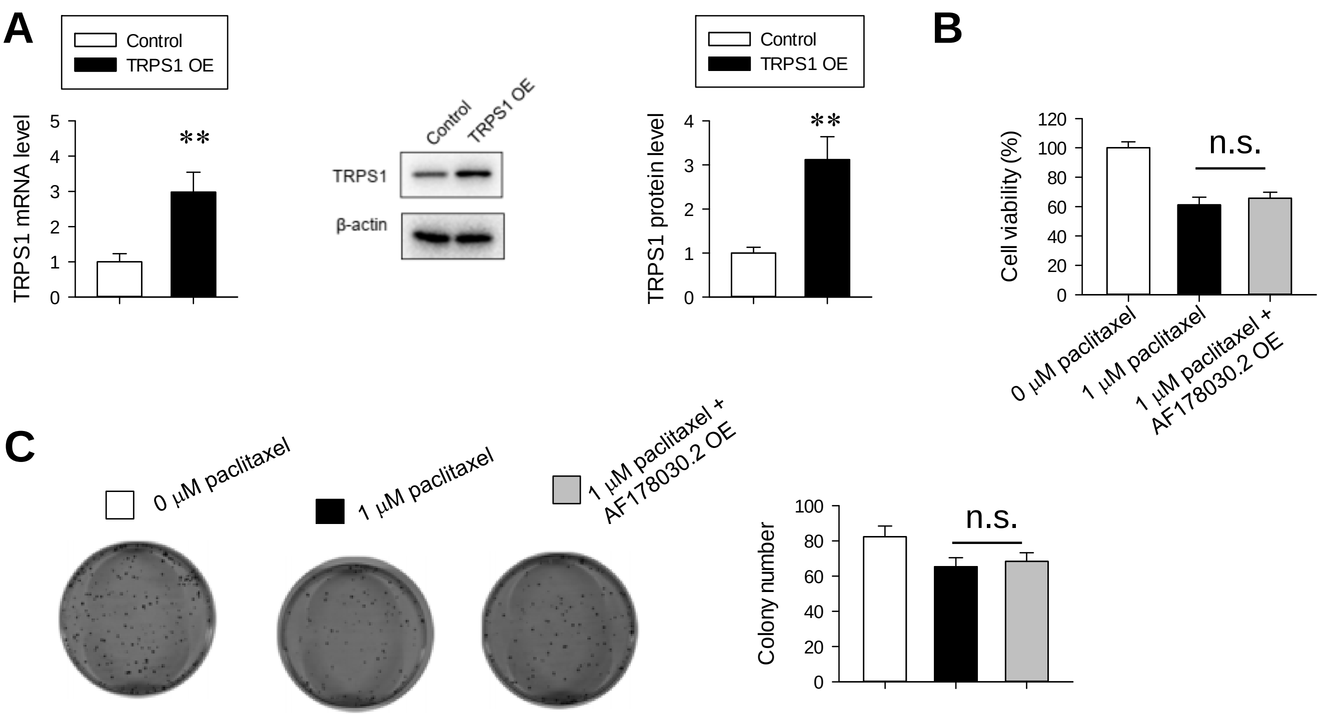

Supplemental Figure 5. Overexpression of TRPS1 eliminated the effect of AF178030.2 on the sensitivity of TNBC cells to paclitaxel. A. Quantitative RT-PCR and Western blot results showing the overexpression of TRPS1 in MDA-MB-436 cells. ***$P<0.01$* versus control group. B. MTT assay showing the effect of TRPS1 overexpression on the cell viability. C. Colony formation assay showing the colony number after TRPS1 overexpression. n=5.
